# Cell migration inhibition activity of a non-RGD disintegrin from *Crotalus durissus collilineatus* venom

**DOI:** 10.1186/s40409-018-0167-6

**Published:** 2018-10-20

**Authors:** Isadora Sousa de Oliveira, Rafaella Varzoni Manzini, Isabela Gobbo Ferreira, Iara Aimê Cardoso, Karla de Castro Figueiredo Bordon, Ana Rita Thomazela Machado, Lusânia Maria Greggi Antunes, José Cesar Rosa, Eliane Candiani Arantes

**Affiliations:** 1 School of Pharmaceutical Sciences of Ribeirão Preto, Department of Physics and Chemistry, University of São Paulo, Av. do Café s/n°, Monte Alegre, Ribeirão Preto, SP 14040-903 Brazil; 2 Department of Clinical Analysis, Toxicology and Food Science, School of Pharmaceutical Sciences of Ribeirão Preto, University of São Paulo, Ribeirão Preto, SP Brazil; 3 Protein Chemistry Center and Department of Molecular and Cell Biology and Pathogenic Bioagents, School of Medicine of Ribeirão Preto, University of São Paulo, Ribeirão Preto, SP Brazil

**Keywords:** *Crotalus durissus collilineatus*, Non-RGD disintegrin, Cell migration, Cell adhesion, Human breast cancer, MDA-MB-231

## Abstract

**Background:**

In recent decades, snake venom disintegrins have received special attention due to their potential use in anticancer therapy. Disintegrins are small and cysteine-rich proteins present in snake venoms and can interact with specific integrins to inhibit their activities in cell-cell and cell-ECM interactions. These molecules, known to inhibit platelet aggregation, are also capable of interacting with certain cancer-related integrins, and may interfere in important processes involved in carcinogenesis. Therefore, disintegrin from *Crotalus durissus collilineatus* venom was isolated, structurally characterized and evaluated for its toxicity and ability to interfere with cell proliferation and migration in MDA-MB-231, a human breast cancer cell line.

**Methods:**

Based on previous studies, disintegrin was isolated by FPLC, through two chromatographic steps, both on reversed phase C-18 columns. The isolated disintegrin was structurally characterized by Tris-Tricine-SDS-PAGE, mass spectrometry and N-terminal sequencing. For the functional assays, MTT and wound-healing assays were performed in order to investigate cytotoxicity and effect on cell migration in vitro, respectively.

**Results:**

Disintegrin presented a molecular mass of 7287.4 Da and its amino acid sequence shared similarity with the disintegrin domain of P-II metalloproteases. Using functional assays, the disintegrin showed low cytotoxicity (15% and 17%, at 3 and 6 μg/mL, respectively) after 24 h of incubation and in the wound-healing assay, the disintegrin (3 μg/mL) was able to significantly inhibit cell migration (24%, *p* < 0.05), compared to negative control.

**Conclusion:**

Thus, our results demonstrate that non-RGD disintegrin from *C. d. collilineatus* induces low cytotoxicity and inhibits migration of human breast cancer cells. Therefore, it may be a very useful molecular tool for understanding ECM-cell interaction cancer-related mechanisms involved in an important integrin family that highlights molecular aspects of tumorigenesis. Also, non-RGD disintegrin has potential to serve as an agent in anticancer therapy or adjuvant component combined with other anticancer drugs.

## Background

Disintegrins are low-molecular-mass cysteine-rich peptides found in snake venom that comprise from 40 to 100 amino acid residues [1–3]. In 1987 Huang et al. observed that a small protein isolated from *Trimeresurus gramineus* venom, called “trigramin”, presenting the arginine-glycine-aspartic acid (RGD) domain, was able to inhibit platelet aggregation, by preventing the connection between fibrinogen and platelets stimulated by ADP [3]. The term “disintegrin” was coined by Gould et al. when they demonstrated that the mechanism for inhibition of platelet aggregation was related to interaction between fibrinogen and α_IIb_β_3_ platelet integrins [4].

Most snake venom disintegrins are believed to be released by proteolytic processing of snake venom metalloproteases (SVMP) from the class P-II [1, 5]. Briefly, SVMP can be classified into three classes according to domain composition present in their structures: (i) P-I SVMP only contains a domain of metalloproteases; (ii) P-II SVMP has a metalloprotease domain and a disintegrin domain on its C-terminal tail, that can be released; (iii) and P-III SVMP contains a metalloprotease domain, a disintegrin-like domain and cysteine-rich domain [6].

Similarly to metalloproteases, disintegrins can also be classified according to their structure, considering the numbers of disulfide bonds and amino acid residues: (i) short disintegrins contain 41 to 51 amino acid residues, stabilized by 4 disulfide bonds; (ii) medium-sized disintegrins contain approximately 70 amino acid residues and 6 disulfide bonds; (iii) long disintegrins, with approximately 84 residues and 7 disulfide bonds; (iv) and the last group that comprises homo- and heterodimeric disintegrins, with approximately 67 residues per subunit, 4 intrachain disulfide bonds, in addition to 2 interchain cystine bonds, stabilizing the molecules [7–10].

In general, these molecules carry this name because they have the RGD motif in their primary amino acid sequence, which is capable of binding to integrins [4]. However, due to a mutation or a sequence of up to three mutations, other domains can be generated, also characterizing a disintegrin by inhibiting other types of integrins [2, 11]. The classical RGD domain is capable of blocking α_IIb_β_3_, α_5_β_1_, α_8_β_1_, α_v_β_1_ and α_v_β_3_ integrins, while the KGD domain inhibits integrin α_IIb_β_3_ with high selectivity; WGD domain inhibits α_IIb_β_3_, α_5_β_1,_ and α_v_β_3_ integrins; MGD and VGD domains can affect the α_5_β_1_ integrin function; KTS and RTS domains are inhibitors of α_1_β_1_ integrin; MLD domain targets the integrins α_3_β_1_, α_4_β_1_, α_4_β_7_, α_6_β_1_, α_7_β_1_ and α_9_β_1_; and the adhesive function of α_IIb_β_3_ can be blocked by the MVD domain [12–14]. As an example, we can mention ussuristatin 2 *Agkistrodon ussuriensis* venom [15] and barbourin from *Sistrurus M. barbouri* [16], which lack the classical RGD domain, as well as atrolysin E from *Crotalus atrox*, from P-II SVMP, which possesses the MVD motif [17].

Due to this ability related to integrins, disintegrins also can be used for the therapy of vascular diseases and bacterial infections (α_5_β_1_), autoimmune diseases and inflammation (α_4_β_1_, α_7_β_1_ and α_9_β_1_), thrombosis and acute coronary syndromes (α_IIb_β_3_), rheumatoid arthritis and osteoporosis (α_v_β_3_), tumor angiogenesis (α_1_β_1_ and α_v_β_3_) and metastasis (α_v_β_3_) [2]. Nowadays, there are two drugs approved by the Food and Drug Administration (FDA) whose design is based on two disintegrins isolated from snake venoms. Eptifibatide (Integrilin®) and Tirofiban (Aggrastat®) were approved in 1998 and 1999, respectively, and are used for acute coronary syndromes because they target the α_IIb_β_3_ integrin [18]. Eptifibatide is based on the KGD motif from barbourin, a disintegrin from *Sistrurus miliarius barbourin* [19], while Tirofiban is based on the RGD motif from echistatin, a disintegrin from *Echis carinatus* [20].

Integrins are closely related to the initiation, promotion and progression of tumors and metastasis [21]. Therefore, disintegrins may play an important therapeutic role as a potential anticancer drug, as is the case of contortrostatin [2]. Zhou et al. observed that this disintegrin from *Agkistrodon contortrix contortrix* venom was not able to cause cytotoxicity in MDA-MB-435 cells (human melanoma cells formerly classified as a human breast cancer cell line), and also prevented the binding of these cells to integrins (α_v_β_3_) and extracellular matrix proteins, such as vitronectin and fibronectin, thus inhibiting the adhesion process [22]. There are many other examples of disintegrins that act as anticancer agents, such as saxatilin, from *Gloydius saxatilis*, which is capable of inhibiting the growth of tumors [23], leucurogin, from *Bothrops leucurus*, that has an anti-angiogenic effect [24] and the adinbitor, from *Agkistrodon halys stejneger*, which also inhibits angiogenesis both in vitro and in vivo [25]. Eritostatin, from *Eristicophis macmahoni* venom, was able to inhibit the migration of melanoma cells, an effect mediated by binding of fibronectin to integrins [26], whereas crotatroxin 2, from *Crotalus atrox* venom, inhibited cell migration of breast carcinoma cells [27]. Interestingly, cilengitide, a cyclic mimetic peptide that has affinity for α_v_β_3_ and α_v_β_5_ integrins, displays antiangiogenic action in low amounts. However, in phase III clinical trials, this drug did not show positive results for patients newly diagnosed with glioblastoma [28, 29]. There are also studies that evaluated the effects of disintegrins on individual stages of metastasis, including cell cycle arrest, extravasation and cell migration [21].

Snake toxins, including disintegrins, are attracting more interest in the fields of medicine and biotechnology. Based on that, this study reports the isolation and structural characterization of a non-RGD disintegrin from *Crotalus durissus collilineatus*, as well as its cytotoxic effect and functional role in the migration of MDA-MD-231 human breast cancer cells, a highly metastatic cell line which is triple negative to progesterone and estrogen receptors [30, 31].

## Methods

### Snake venoms

*Crotalus durissus collilineatus* venom was provided by the Serpentarium at the School of Medicine of Ribeirão Preto, University of São Paulo, Brazil, accredited by Brazilian Institute of the Environment and Renewable Natural Resources (IBAMA), and registered under number 1506748, for scientific purposes. All animals were adult and crotamine-negative. The extracted venoms were dried at room temperature for 6 h in a vacuum desiccator and stored at − 20 °C until use.

### Purification of disintegrin

Disintegrin from *C. d. collilineatus* venom was purified through two chromatographic steps carried out in a Fast Protein Liquid Chromatography (FPLC) system (Äkta Purifier UPC 900, GE Healthcare, Uppsala, Sweden). The venom (30 mg) was dispersed in 0.1% trifluoroacetic acid (TFA, solution A) and 1% formic acid, and centrifuged at 13,000 × *g* at 4 °C for 10 min. The supernatant was fractionated on a C18 column (250 × 10 mm, 5 μm particles, 300 Å, Phenomenex, Torrence, CA, USA) at a flow rate of 5 mL/min, using the concentration gradient described by Calvete et al. [32]. The second step was performed on another C18 column (250 × 4.6 mm, 3.6 μm particles, Phenomenex, Torrence, CA, USA) at a flow rate of 1 mL/min and the proteins were eluted using a segmented concentration gradient from 6.3 to 100% of solution B (80% acetonitrile, ACN, in 0.1% TFA). In both steps, the protein elution was monitored by absorbance at 214 nm. Fractions of interest were collected, frozen and lyophilized for further analysis.

### Mass determination

The fractions of interest were analyzed by Tris-Tricine-SDS-PAGE (16.5%) [33] under reducing conditions. The molecular mass markers of 97.0–14.4 kDa (17–0446-01, GE Healthcare) and 26.6–1.06 kDa (M3546-1VL, Sigma-Aldrich, Saint Louis, MO, USA) were used. Proteins were stained with 0.2% *Coomassie Brilliant Blue G-250* (Sigma).

Also, the accurate molecular mass was determined by matrix-assisted laser desorption/ionization (MALDI) with time of flight (TOF) analyzer and Smartbeam II laser, an ultrafleXtreme instrument with the software FlexControl, version 3.3 (Bruker Daltonics GmbH, Leipzig, Germany) for the acquisition of data. The parameters employed to obtain the data were 500 laser shots per spectrum, 1000 Hz laser frequency and the instrument operating in positive reflected mode, within a range of 5 to 50 kDa, according to the manufacturer’s instructions. UltrafleXtreme instrument was calibrated using a mixture of peptides (Peptide calibration standard, NC9846988) and proteins (Protein calibration standard I, NC0239984, and Protein calibration standard II, NC0416074) from Bruker Daltonics. A saturated solution of α-cyano-4-hydroxycinnamic acid (α-CHCA) matrix was prepared in ACN and 0.1% TFA (V/V), at the ratio of 1:1 (V/V). Data analysis was performed by the software FlexAnalysis, version 3.3 (Bruker Daltonics GmbH, Leipzig, Germany).

### Protein identification

For protein identification, the fraction of interest was submitted to N-terminal and MS/MS sequencings.

N-terminal sequencing was performed by the Edman degradation method [34], using the automated protein sequencer model PPSQ-33A (Shimadzu, Kyoto, Japan), according to the manufacturer’s instructions. The similarity of the amino acid sequences obtained in relation to sequences already deposited in databanks was evaluated by the Basic Local Alignment Search Tool (BLAST) [35].

The fraction of interest was reduced, alkylated, digested with sequencing grade porcine pancreatic trypsin and applied on a reversed phase C18 column (0.075 × 100 mm, 1.7 μm particles, 130 Å, Waters, Manchester, UK) for ultra-performance liquid chromatography (UPLC) coupled to electrospray (ESI) mass spectrometer, with quadrupole-time of flight (Q-TOF) analyzer (API-US, Waters, Manchester, UK). Data were interpreted with a licensed version of the MASCOT program against databank protein sequence deposited in the SwissProt (554,241 sequences, 198,410,167 amino acid residues) database and a databank of protein sequences filtered for snake venoms (27,207 sequences, 10,540,234 amino acid residues), generated from UniProt Knowledgebase (UniProtKB) [36]. Precursor mass tolerance was 1.2 Da and MS/MS mass tolerance was set to ±0.8 Da, carbamidomethyl cysteine was set as fixed modification and oxidation of methionine as variable modification.

### Functional assays

To verify whether the protein has cytotoxic activity and interferes with cell migration, two functional tests were carried out: cell viability assay by MTT and cell migration assay by wound healing in monoculture (MDA-MB-231). Human breast cancer cell line (MDA-MB-231), obtained from American Type Culture Collection (ATCC, Cat No. HTB-26), was cultured in Dulbecco’s Modified Eagle’s Medium (DMEM) medium supplemented with 4 mM L-glutamine, 10% fetal bovine serum and 1% antibiotics (streptomycin, gentamycin and neomycin). Cells were maintained under standard conditions at 37 °C, 5% CO_2_ and 95% humidity in a Forma Series II stove, Water Jacket CO_2_ Incubator (ThermoFisher Scientific, Waltham, Massachusetts, USA). All the experiments were conducted between the third and the eighth cell passage.

MDA-MB-231 cells were submitted to 3-[4, 5-dimethylthiazol-2-yl]-2, 5 diphenyl tetrazolium bromide (MTT) assay according to Mosmann [37]. Initially, MDA-MB-231 cells were seeded in 96-well culture plates at the density of 1 × 10^4^ cells per well. Subsequently, the plates were incubated at 37 °C in 5% CO_2_ for 24 h. After the incubation time, the cells were treated with the toxin at concentrations of 0.75; 1.50; 3.00; 6,00 μg/mL. Phosphate buffered saline (PBS) was used as negative control, and methyl methanesulfonate (MMS) as positive control (300 μM or 33,039 mg/L). After 24 h of treatment, 20 μL of MTT solution (5 mg/mL) was diluted in PBS and added to each well and the plate was incubated for 3 h. After the incubation time, the plates were centrifuged for 5 min at 1027 × *g*, the supernatant removed and 200 μL of dimethyl sulfoxide (DMSO) added. Absorbance reading was done at 570 nm using a microplate reader (Biotek EL800 - Winooski, USA). The percentage of cell viability was obtained by the ratio between the absorbance of each treatment well and the absorbance of the wells of the negative control cultures, with cell viability considered 100%.

For the wound healing assay, 3.0 μg/mL of disintegrin was used, and the assay was performed according to Liang, Park and Guan [38]. MDA-MB-231 cells (1.5 × 10^5^ cells/well) were grown to 90% confluence on a 12-well plate. Then, with the aid of a sterile tip, a cicatrix was performed on the adherent monolayer of tumor cells. The culture medium was changed to remove the cell debris and a new culture medium added containing the lowest concentration (3.0 μg/mL) of disintegrin which demonstrated effects on the cell line in the MTT assay and PBS as CN. Images of the wounds were recorded with a camera coupled to the inverted microscope at times 0, 4, 8 and 24 h of incubation. The mean distance of the internal area was determined as the percentage migration using the software AxionVision according to the following equation:

$$ Cell\ migration\ \left(\%\right)=\frac{\left( At=0h\right)-\left( At=24h\right)}{\left( At=0h\right)}\times 100 $$

All the results were expressed as the mean ± standard deviation (SD). Data from three independent experiments (*n* = 3) were statistically analyzed using the software GraphPad Prism 5 (La Jolla, CA, USA). After assessing normality of variable distributions using the Kolmogorov–Smirnov test, experimental data were analyzed using one-way analysis of variance (ANOVA) followed by Dunnett’s test. Statistical significance was considered at *p* < 0.05.

## Results

### Purification and identification of non-RGD disintegrin

**Fig. 1:**
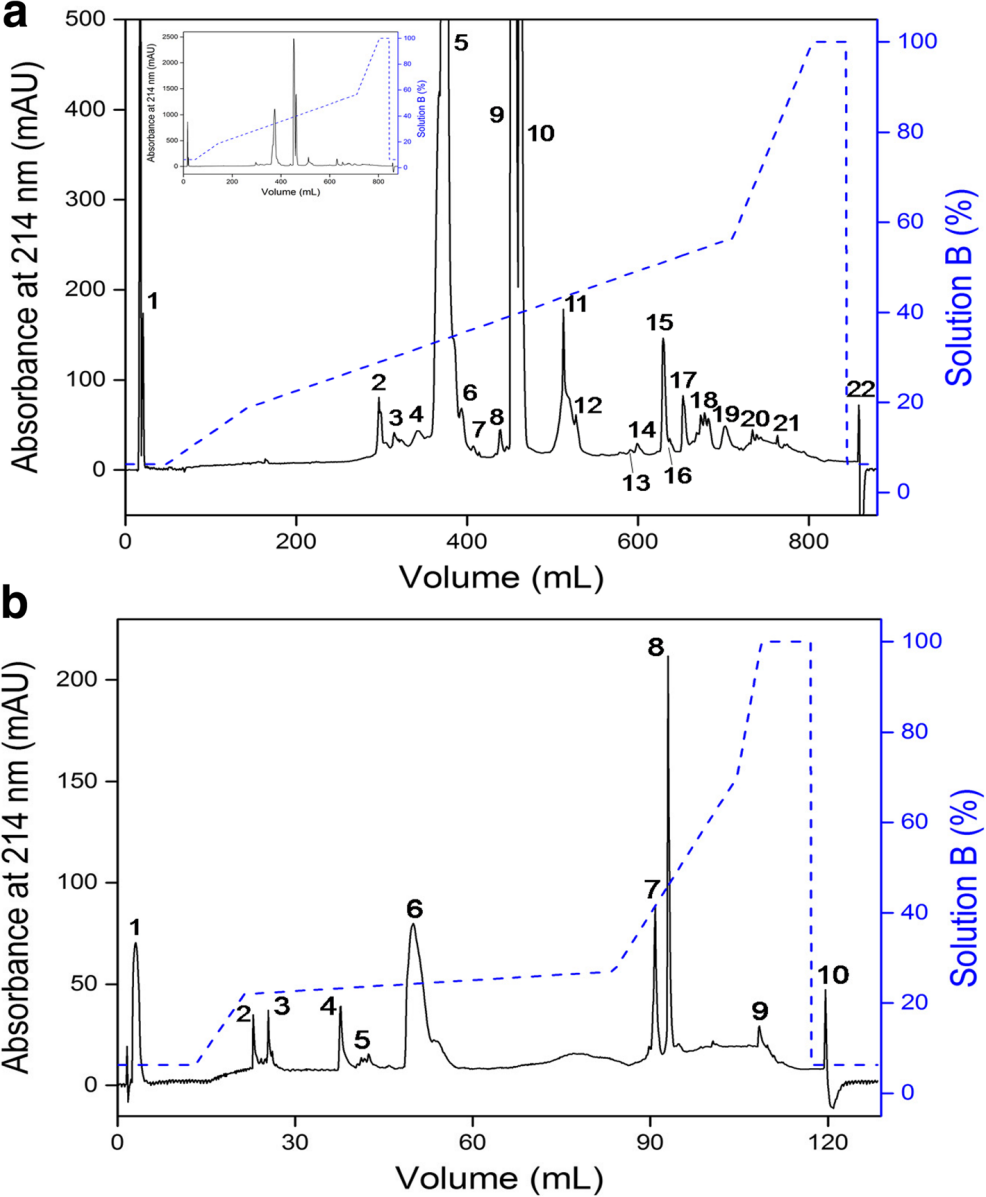
Chromatographic profiles of non-RGD disintegrin from *C. d. collilineatus* venom using RP-FPLC system. **a** *C. d. collilineatus* venom (30 mg) was applied on a C18 column (250 × 10 mm, 5 μm particles, 300 Å), at a flow rate of 5 mL/min and (**b**) Fraction 2 (200 μg) on a C18 column (250 × 4.6 mm, 3.6 μm particles), at a flow rate of 0.5 mL/min. Elution in both chromatograms was carried out in a segmented concentration gradient from 6.3 to 100% of solution B (80% ACN in 0.1% TFA, represented by the blue dashed line) and absorbance was monitored at 214 nm. Inset panel – whole chromatographic profile without magnification

Purification of disintegrin was performed in two steps, both on reversed phase C18 columns. The first step resulted in 22 fractions (Fig. 1a) and Fraction 2 was chosen for further analysis. In order to isolate the disintegrin, Fraction 2 was refractionated by reversed phase chromatography on a FPLC system with a segmented acetonitrile gradient represented by the dashed line in blue (Fig. 1b). The final chromatographic profile presented 10 fractions, with Fraction 6 being the major and most probable fraction where the disintegrin may have eluted, representing 0.43% of the total venom (Table 1).

**Table 1:** Protein recovery (%) during the purification procedure of disintegrin from *Crotalus durissus collilineatus* venom

Sample	Purification steps	^a^Protein recovery (%)
*Cdc* venom	Venom solubilization	100.0
Fraction 2	RP-FPLC on a C-18 column (1st chromatography)	0.94
Fraction 6	RP-FPLC on a C-18 column (2nd chromatography)	0.43

^a^The recovery percentage of each peak was calculated by the software Unicorn 5.2 (GE Healthcare) using the relation between the area under the curve of absorbance at 214 nm of the corresponding peak and the sum of the areas of all eluted peaks

**Fig. 2:**
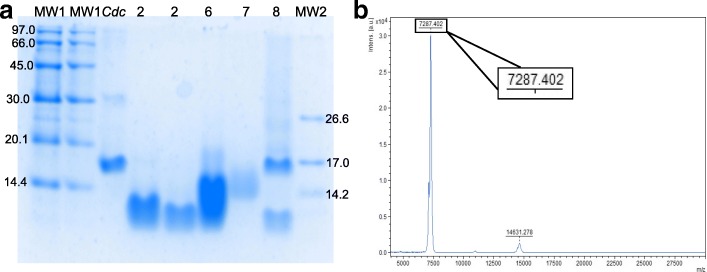
Isolation profile of non-RGD disintegrin from *C. d. collilineatus* venom. **a** Tris-tricine-SDS-PAGE (16.5%), in non-reduced conditions and the gel was stained with 0.2% *Coomassie Brilliant Blue G-250*. MW1 – molecular mass (97.0–14.4 kDa); *Cdc* – *C. d. collilineatus* venom; 2 – Fraction 2 from RP-FPLC first step; 6–8 – fractions 6, 7 and 8, respectively, from RP-FPLC second step; MW2 – molecular mass (26.6–1.06 kDa). **b** Mass spectrum of Fraction 6 eluted from RP-FPLC second step obtained by MALDI-TOF (positive linear mode) using α-cyano-4-hydroxycinnamic acid (α-CHCA) matrix

The fractions of interest were analyzed by Tris-Tricine-SDS-PAGE (16.5%). The electrophoretic profile revealed that Fraction 2 exhibits a protein band of apparent 7 kDa molecular mass and some contaminants (Fig. 2a). After the second chromatographic step, it was possible to observe in Fraction 6 a single band of nearly 7 kDa corresponding to disintegrin. Thus, it was possible to separate disintegrin from its contaminants of approximately 14 kDa, which eluted in fractions 7 and 8. The molecular mass of the peptide present in Fraction 6 was determined by MALDI-TOF as 7287.402 Da (Fig. 2b).

**Fig. 3:**
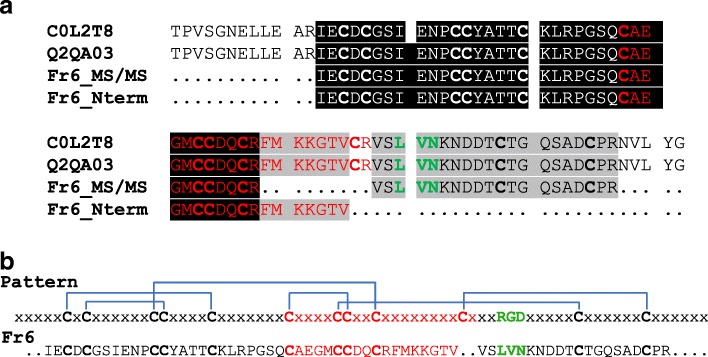
Alignment and primary structure of disintegrins. **a** Sequence alignment between *C. d. collilineatus* (C0L2T8) and *C. d. durissus* (Q2QA03) disintegrins, peptides determined by mass spectrometry analysis (Fr6_MS/MS) and sequence obtained by Edman degradation (Fr6_Nterm) of Fraction 6. **b** Comparison between the classical RGD disintegrin structure pattern and the determined primary structure of Fraction 6 (LVN disintegrin). Bold – cysteines ©; Black box – consensus of all data sequence; Gray box – consensus of three data sequence; Red – pattern signature of disintegrin domain involving five conserved cysteines forming disulfide bonds; Green - RGD motif site; Blue – disulfide bond pattern of disintegrin structure; x – any amino acid residue

The first 43 amino acid residues of the N-terminal region of the Fraction 6 were sequenced by Edman degradation and shared identity with others disintegrins (Fig. 3a). In addition, Fraction 6 was digested with trypsin and submitted to MS/MS sequencing. Data from obtained sequences are shown in Table 2.

**Table 2:** Identification of non-RGD disintegrin peptides by MS/MS sequencing

^a^MS/MS	Peptide			Protein		
	m/z	Z	Score	Access	Specie	Score
NDDTCTGQSADCPR	798.75	2	79	Q2QA03	*C. d. durissus*	347
LRPGSQCAEGMCCDQCR	695.23	3	64			
VSLVNKNDDTCTGQSADCPR	746.27	3	100			
IECDCGSIENPCCYATTCK	1169.36	2	105			

^a^MS/MS: peptides identified by mass spectrometry using ESI

As to the in silico analysis, it was observed that these sequences shared similarity with the disintegrin domain (amino acid residues 397 to 478) of metalloproteases PII from *C. d. collilineatus* (C0L2T8) and *C. d. durissus* (Q2QA03), including the conservation of cysteine amino acid residues (Fig. 3a). Comparing the amino acid residues obtained from Fraction 6 with the primary sequences of both disintegrins, there was 76.8% coverage of the total sequence using Edman degradation and mass spectrometry procedures. In addition, these results show that the disintegrin of this study is a non-RGD disintegrin. The classic RGD motif, the conserved cysteine amino acid residues and the disulfide bond pattern commonly observed in disintegrins, as well as the determined sequence of Fraction 6 (LVN disintegrin) are shown in Fig. 3b for comparison purposes.

### Functional assays with non-RGD disintegrin

**Fig. 4:**
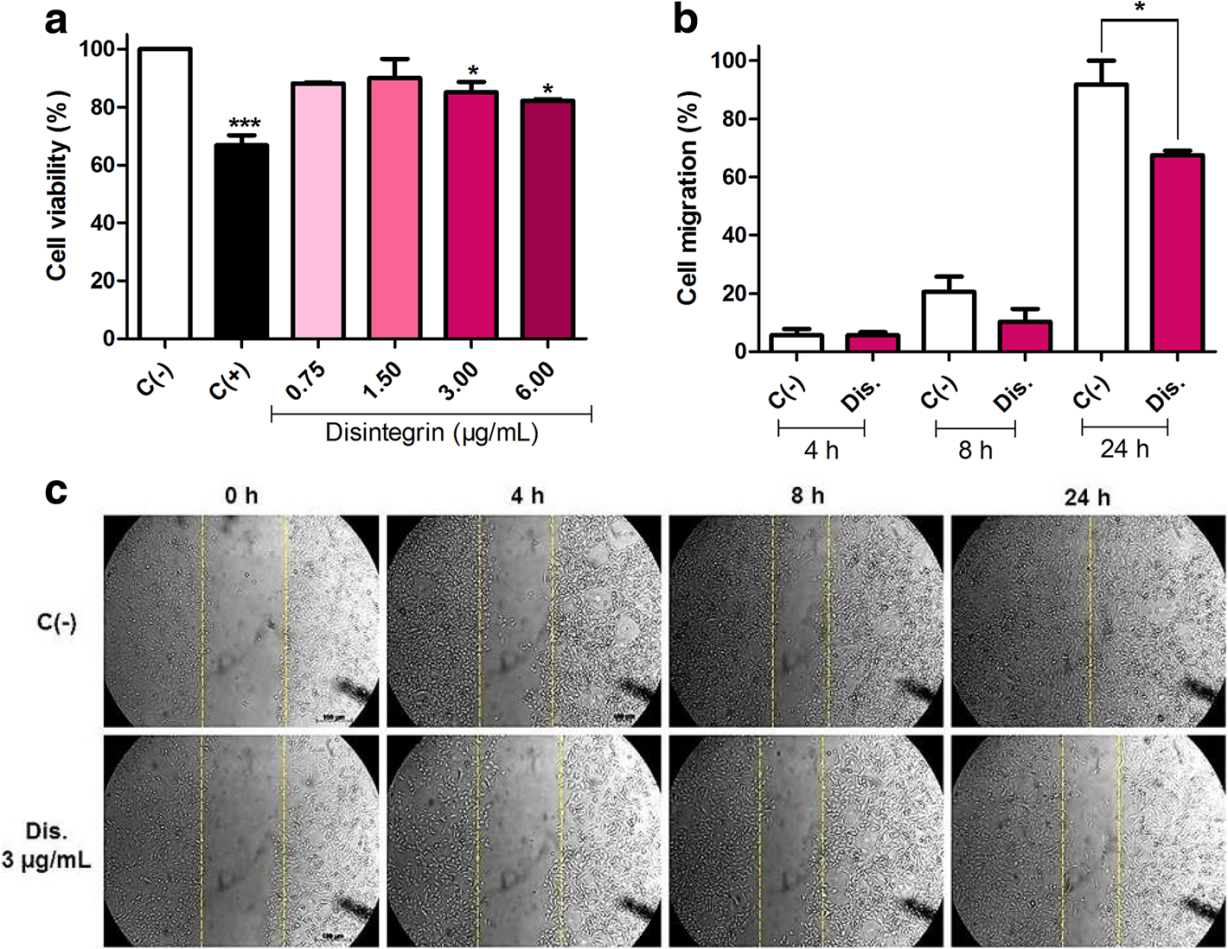
Functional assays with MDA-MB-231 cells. **a** Cell viability (%) after 24 h of treatment with disintegrin (0.75–6.00 μg/mL). C (−) – negative control (PBS); C (+) – positive control (MMS – 300 μM). The results were analyzed by ANOVA, followed by Dunett’s post-hoc test (*p < 0.05). **b** and **c** Cell migration after different times (0, 4, 8 and 24 h) of treatment with non-RGD disintegrin (3 μg/mL). C (−) – negative control (PBS). The dashed yellow lines delimit the region where no cell growth is observed. Data (*n* = 3) are presented as mean ± SD, which were analyzed by Student’s *t* test (*p < 0.05)

Cell viability of MDA-MB-231 was determined in the presence of disintegrin; it was observed that the concentrations of 3 and 6 μg/mL of disintegrin significantly decreased cell viability in approximately 15% and 17%, respectively (Fig. 4a). Considering that disintegrin presented low yield in the purification steps (0.43%, Table 1) and that the two concentrations did not differ statistically (*t* test - data not shown), we chose to use the concentration of 3 μg/mL in the wound healing assay.

The cell migration assay based on wound healing showed that disintegrin was able to significantly inhibit cell migration of MDA-MB-231 (24%, *p* < 0.05 compared to negative control), after 24 h of incubation (Fig. 4b and c).

## Discussion

In the present study, we aimed to perform the isolation, structural and functional characterization of a non-RGD disintegrin from *C. d. collilineatus* venom, which had never before been described. Only two chromatographic steps were sufficient to isolate non-RGD disintegrin. The choice of the methodology was based on previous venomic studies that had reported the presence of disintegrin in this subspecies venoms [39, 40].

Pure non-RGD disintegrin corresponds to 0.43% of soluble venom. This protein recovery is within the range of 0.4–0.5% of disintegrins in the venom of this subspecies determined by proteomic techniques [40]. Due to low amounts of disintegrins in snake venoms [40, 41], is necessary to produce recombinant disintegrins as similar to contortrostatin [42], r-colombistatins 2, 3, and 4 [43] and r-Cam-dis [44], among others.

Non-RGD disintegrin from *C. d. collilineatus* presents 7287.4 Da, as determined by MALDI-TOF, and it is similar to the molecular mass of others snake venom disintegrins, such as tzabcanin (7.1 kDa) [45], disintegrin from *C. simus* (7.1 kDa) [46] and proteomic data of *C. d. collilineatus* [39, 40].

As to the primary sequence obtained through Edman degradation and mass spectrometry procedures, *Cdc* non-RGD disintegrin isolated in this study is absent from the classical RGD motif or any other motif previously observed in this protein family or glutamate-cysteine-aspartate (ECD) motif which characterize disintegrin-like proteins that may inhibit tumor progression [47]. In the same position of this domain, this toxin presents a triad of amino acids LVN, which was found in disintegrins of *C. d. collilineatus* by transcriptome techniques [48], as well as in the disintegrin domains of P-II SVMP of *C. d. collilineatus* (C0L2T8) and *C. atrox* (Q2QA03). On the other hand, these disintegrins, as well as that of our study, present the disulfide bond pattern of this protein family, another signature that characterizes disintegrins. This pattern of disulfide bonds has already been well reported and is closely related to the formation of the protein loop and activity. If disintegrins are reduced and alkylated their biological activity is decreased [49–53].

Disintegrins are known to be potent inhibitors of platelet aggregation, and some drugs based on its structure were developed and approved by the FDA [54], such as Eptifibatide [19] and Tirofiban [20]. The main mechanism for such inhibition is that the disintegrins bind to the α_IIb_β_3_ platelet integrins, preventing their binding to fibrinogen and consequently inhibiting platelet aggregation [3]. Knowing that the integrins of platelets (α_IIb_β_3_) and breast cancer cells (α_v_β_3_) are similar, disintegrins are considered candidates to be anticancer agents [2]. Integrins are transmembrane proteins, that are part of cell adhesion molecules (CAM) group, and are formed by two subunits, α, of approximately 120 to 180 kDa, and β, of approximately 90 to 110 kDa, non-covalently linked [55]. They are able to perform the cell-cell and cell-ECM interactions [56], and are essential for the development of tumor cells [57].

Since disintegrins are considered candidates to be agents of anticancer therapy, we performed cytotoxicity and cell migration assays on metastatic breast cancer cells, namely MDA-MB-231 cells. This cell line is able to express low levels of the subunits α_2_ and β_5_ and moderate levels of α_v_, α_3_ and β_1_ integrins [58]. Taherian et al. demonstrated that MDA-MB-231 cells express higher levels of β_5_ and α_v_β_5_ integrins when compared with non-breast cancer cell line (Hek-293) [59]. Our results show that non-RGD disintegrin from *C. d. colilineatus* venom presents low cytotoxicity, although its toxicity increases with the concentration, having a dose-response effect. As in our results, the tzabcanin also showed dose-dependent toxicity against human malignant melanoma (A-357) and human colorectal adenocarcinoma (Colo-205) cell lines, but the viabilities of human breast adenocarcinoma (MCF-7) and human lung adenocarcinoma (A-549) cell lines were not affected [45, 60]. Lebein inhibited the viability of human colon adenocarcinoma (HT29, LS174 and HCT116) and melanoma (SK-MEL-28 and LU-1205) cell lines [28, 61].

The cell migration assay showed that *Cdc* non-RGD disintegrin was able to inhibit the migration of MDA-MB-231 cells after 24 h of incubation, demonstrating that it is a possible anticancer agent with potential to inhibit the formation of breast cancer tumors. Similar results were obtained from tzabcanin, which inhibited cell migration of A-375 and A-549 cell lines [60], r-Viridistatin 2, from *Crotalus viridis viridis,* and r-mojastin 1, from *Crotalus scutulatus scutulatus,* that inhibited the migration of human pancreatic adenocarcinoma cancer cells (BXPC-3) [62].

In addition, considering the potential of disintegrins as anticancer therapeutic agents, they can also be used in combination with known chemotherapeutics. For example, echistatin combined with cisplatin (CDDP) was capable of increasing the expression of nuclear factor kappa B (NFκB), caspase-9 and caspase-3, leading to increased apoptosis in MDA-MB-231 cells. These data suggest that the combination of this disintegrin with CDDP may serve as a new type of anticancer therapy [63].

## Conclusion

In this study, we described the isolation and characterization of the first disintegrin with an LVN motif from *C. d. collilineatus* venom. The studies performed on the human breast cancer MDA-MB-231 cell line demonstrate that this disintegrin is able to inhibit cell migration, without severely affecting cell viability. Therefore, it may be a very useful molecular tool for understanding ECM-cell interaction cancer-related mechanisms involved important family of integrin that would elucidate molecular aspects of tumorigenesis. Also, this non-RGD disintegrin has potential to serve as an agent in anticancer therapy or adjuvant component combined with other anti-cancer drugs.

## Abbreviations

ACN: Acetonitrile
ADP: Adenosine diphosphate
ANOVA: Analysis of variance
ATCC: American Type Culture Collection
BLAST: Basic Local Alignment Search Tool
CAM: Cell adhesion molecules
CDDP: Cisplatin
DMEM: Dulbecco’s Modified Eagle’s Medium
DMSO: Dimethyl sulfoxide
ECD: Glutamate-cysteine-aspartate
ECM: Extracellular matrix
ESI: Electrospray
FDA: Food and Drug Administration
FPLC: Fast Protein Liquid Chromatography
IBAMA: Brazilian Institute of the Environment and Renewable Natural Resources
KGD: Lysine-glycine-aspartic acid
LVN: Leucine-valine-asparagine
MALDI: Matrix-assisted laser desorption/ionization
MDC: Metalloprotease/disintegrin/cysteine-rich
MGD: Methionine-glycine-aspartic acid
MLD: Methionine-leucine-aspartic acid
MMS: Methyl methanesulfonate
MS/MS: Tandem mass spectrometry
MTT: 3-[4, 5-dimethylthiazol-2-yl]-2, 5 diphenyl tetrazolium bromide
MVD: Methionine-valine-aspartic acid
PBS: Phosphate buffered saline
Q-TOF: quadrupole-time of flight
RGD: Arginine-glycine-aspartic acid
SDS-PAGE: Sodium dodecyl sulfate polyacrylamide gel electrophoresis
SVMP: Snake venom metalloproteases
TFA: Trifluoroacetic acid
TOF: Time of flight
UPLC: Ultra-performance liquid chromatography
VGD: Valine-glycine-aspartic acid
WGD: Tryptophan-glycine-aspartic acid
α-CHCA: α-cyano-4-hydroxycinnamic acid
